# Exercise is medicine

**DOI:** 10.3389/fnagi.2023.1129221

**Published:** 2023-01-30

**Authors:** Jiahui Li, Huaide Qiu, Jianan Li

**Affiliations:** ^1^Rehabilitation Medical Center, The First Affiliated Hospital of Nanjing Medical University, Nanjing, China; ^2^Faculty of Rehabilitation Science, Nanjing Normal University of Special Education, Nanjing, China

**Keywords:** exercise, medicine, functioning, health, physical activity

## Introduction

The increase in the population of the elderly and the prevalence of chronic diseases has resulted in a growing number of people living with disabilities (Cieza et al., 2021). In such cases, mortality and morbidity cannot adequately describe the clinical outcomes of most health conditions. Hence, in 2016, the World Health Organization (WHO) proposed functioning as the third clinical outcome indicator (Stucki and Bickenbach, 2017). Exercise is an important modality for improving functioning and health conditions. The American College of Sports Medicine and the American Medical Association have suggested that “exercise is medicine^®^” (Lobelo et al., 2014), and this marks a new direction in healthcare. Exercise plays an important role in the prevention, treatment, and rehabilitation of diseases, and is the basis of active and universal health. However, the initiative that “exercise is medicine” has been disputed. This article discusses this statement from the perspective of the proposal of “exercise is medicine” initiative, consequences of exercise in health, and considerations of exercise.

## The proposal of “exercise is medicine” initiative

### Lack of exercise: A global problem

Lack of exercise is a health concern worldwide. According to WHO, 31% of the world’s population does not attain the minimum required level of physical activity (Kohl et al., 2012). This unhealthy lifestyle is the fourth leading cause of death worldwide with approximately 3.2 million deaths annually (Hall et al., 2021). This situation is continuously worsening and poses a substantial burden on health systems and societies.

A considerable portion of health conditions can be attributed to physical inactivity. Sedentary behavior and physical inactivity are the leading risk factors for cardiovascular disease and all-cause mortality (Lavie et al., 2019). Compared with people who have previously been physically active, those who are inactive have a higher risk of developing neurological diseases (Pedersen and Saltin, 2015; Prior and Suskin, 2018; Kim et al., 2019). A systematic review revealed that the incidence of osteoarthritis among people who engaged in running exercisers was lower than the incidence in those who do not exercise; furthermore, lack of exercise is associated with a high risk of sports injuries and musculoskeletal diseases (Alentorn-Geli et al., 2017). Increasing evidence suggests that exercise can play an important role in cancer prevention; people who are physically inactive have a higher cancer morbidity than those who exercise regularly (Hojman et al., 2018; Wang and Zhou, 2021). In addition, lack of exercise can accelerate aging, resulting in a rapid decline in functioning (Angulo et al., 2020).

### Role of exercise in healthcare

As incurable chronic diseases have become a major health concern, an increasing number of people experience a decline in functioning. It is widely accepted that functioning is the basis of health, and exercise is crucial for maintaining and improving function. This situation drives the demand for rehabilitation services. Rehabilitation is to take specific interventions against patients’ disabilities in order to restore their functioning, reestablish their health, and reintegrate them into the society. These interventions are predominantly exercise training and relevant physical activity. However, exercise is rarely used as medical advice in other clinical settings. The role of exercise in healthcare should be highlighted.

### The presentation of “exercise is medicine” initiative

Recognizing that many chronic diseases are closely related to poor lifestyle habits and that exercise plays a role in a variety of health conditions, the American College of Sports Medicine and the American Medical Association suggested that “exercise is medicine^®^” (Lobelo et al., 2014; Li and Laher, 2020). Subsequently, a multinational collaboration on “exercise is medicine^®^” began and this initiative was centered on global awareness. In this initiative, it was suggested that patients’ level of physical activity be added to their medical records, and behavioral physical activity counseling should be provided through a clinical decision support system. Specifically, the exercise situation of each patient should be considered as a vital sign in each visit, then healthcare workers should provide professional physical activity guidance for patients’ exercise situation and health needs. This initiative emphasized that exercise should be used as medical advice in clinical settings. Hence it is not just improving patients’ exercise awareness that is significant, but more so the formation of normative medical work for exercise guidance and the healthcare workers’ awareness of exercise effects in health.

## Consequences of exercise in health

Exercise acts as a form of medicine in multiple ways. First, exercise changes sedentary behavior, reduces risk factors, and enhances immunity, thus functioning as a form of prophylaxis. Second, appropriate exercise can prevent or alleviate dysfunction after the onset of disease and prevent disease recurrence. That is, exercise plays an important role in primary, secondary, and tertiary prevention of different health conditions.

### Neurological diseases

For neurological diseases, including stroke, dementia, Parkinson’s disease, and multiple sclerosis, exercise can maintain or improve brain metabolism and favorably modify some risk factors, such as hypertension, hyperlipidemia, obesity, excessive alcohol consumption, and tobacco use (Pedersen and Saltin, 2015; Prior and Suskin, 2018; Kim et al., 2019). Exercise can also decrease complications and improve functioning, including motor function, cognitive function, and activities of daily living after the onset of diseases.

### Musculoskeletal diseases

Aging leads to decreased muscle mass and muscle strength, which is also known as sarcopenia (Cruz-Jentoft et al., 2019). Currently, no pharmacological agents have proven effective for the management of sarcopenia. However, exercise, whether resistance training or aerobic training, balances and even enhances muscle function in elderly patients with muscle failure (Papadopoulou, 2020). Exercise can improve bone density, reduce anti-inflammatory response, promote cartilage metabolism, and enhance muscle strength and joint stability. Patients receiving traditional treatment are often prescribed bed rest, which decreases their motor function. Therefore, the development of an appropriate exercise program is crucial for treatment planning, especially for hospitalized patients.

### Cardiopulmonary diseases

Heart failure was previously a contraindication for exercise. However, as science evolves, exercise has become a powerful treatment for heart failure (Cornelis et al., 2016). Regular exercise can improve cardiovascular capacity, increase the threshold for angina, promote coronary plaque regression, reduce endothelial damage, and promote the growth of new coronary collateral vessels. Regular exercise is also effective in lowering blood pressure and preventing hypertension owing to changes in endothelial function and the sympathetic nervous system. Regular exercise has been demonstrated to decrease respiratory symptoms (Armstrong and Vogiatzis, 2019).

### Cancer

Increasing evidence suggests that exercise can play an important role in cancer prevention and treatment by reducing cancer incidence and inhibiting tumor growth (Hojman et al., 2018; Wang and Zhou, 2021). It has been shown that physical activity is associated with a lower risk of cancer recurrence, especially in breast and colorectal cancer (Idorn and Thor Straten, 2017). Exercise can increase the recruitment and infiltration of natural killer (NK) cells, which can kill cancer cells (Idorn and Hojman, 2016). Cancer and its treatment often lead to functional disorders, such as motor dysfunction, cardiopulmonary dysfunction, and mental and psychological dysfunction. The inclusion of exercise intervention in cancer treatment plans can also improve concomitant symptoms, alleviate the side effects of cancer treatment, and enhance clinical therapeutic effects. Therefore, cancer treatment plans should no longer be limited to clinical treatment but should be extended to comprehensive medical services and health management for patients.

### Enhanced recovery after surgery

It was assumed that patients undergoing surgery should have good bed rest. However, as the concept of enhanced recovery after surgery (ERAS) was proposed, people realized the importance of early mobilization to help patients recover their function as quickly as possible (Ljungqvist et al., 2017). The approach to perioperative care must be multimodal and include pain, anesthesia, fluid therapy, nutrition, and mobilization management. Early mobilization can promote the recovery of respiratory, gastrointestinal, musculoskeletal, and other multi-system functions and is beneficial for the prevention of pulmonary infections, pressure sores, deep vein thrombosis of the lower extremities, and other complications. It is recommended that patients start ambulating on the first postoperative day, establish a daily exercise target, and gradually increase the amount of exercise.

### COVID-19

The role of exercise in the coronavirus disease 2019 (COVID-19) pandemic has raised awareness of the benefits of exercise in communicable diseases and has brought new insights into the medical field. Nutrition and appropriate activities are the key means of COVID-19 prevention, treatment, and rehabilitation. Exercise can improve immunosurveillance, resist COVID-19 infection, reduce symptoms, promote recovery, and play a role at the three levels of prevention (Nieman, 2021). Patients with mild COVID-19 can undergo respiratory training, mild aerobic training, and traditional Chinese exercises. Prone position ventilation is currently widely applied for patients with severe COVID-19, and appropriate breathing training and activity training interventions are also important. Many people with COVID-19 develop a series of symptoms once the acute phase of the disease is over, which is known as chronic COVID (Crook et al., 2021). The symptoms include fatigue, dyspnea, “brain fog,” muscle pain, cardiac abnormalities, and autonomic dysfunction. This condition is increasingly affecting a large number of people as the pandemic evolves, posing a huge global challenge. It is important to develop a long-term effective management strategy for long-term COVID. Exercise has been shown to improve many symptoms and reduce the long-term effects of COVID-19 and is recommended as the focus of the management strategy for COVID (Jimeno-Almazán et al., 2021). In addition, a long COVID has places increasing the demand for outpatient rehabilitation. The development of COVID rehabilitation and telehealth will make it possible for more people with long COVID to have access to professional exercise guidance.

### Others

Regarding age-related disabilities, exercise can reduce aging-associated oxidative damage and chronic inflammation, increase autophagy, and improve mitochondrial function, thus maintaining or improving functioning (Angulo et al., 2020).

A study involving more than 1.2 million people indicated that exercise was associated with a low mental health burden (Chekroud et al., 2018). People who exercised reported better mental health than those who did not, regardless of the exercise type they chose, even in people with psychological problems, such as stress, and those with psychiatric diseases, such as depression.

## Considerations of exercise

With the intensification of social aging and less active exercise for most people, global health conditions are becoming increasingly acute. The “exercise is medicine^®^” initiative is a call for early and active investment in health through exercise. In this context, active health has become a modern concept. In China, the Ministry of Science and Technology took “active health and aging technology response” as the theme of Key Special Projects in recent years, which indicated the increasing attention to the health conditions and active health.

The implementation of the “exercise is medicine” solution involves several modules, including the clinical module, community module, and active health technology (Lobelo et al., 2014). Patients can obtain a comprehensive assessment and physical activity prescription in the clinical module, then practice self-management according to the prescriptions in the community module. This process enables the inclusion of exercise in medical delivery and encourages patients to start exercising. Meanwhile, active health technology allows patients to exercise more effectively. The development of exercise equipment, including athletic footwear and apparel, enables people to have a good exercise experience and reduces the probability of exercise injuries to some extent. The latest progress in technology, including rehabilitation robotics, wearable devices, virtual reality, artificial intelligence, and smart telemedicine, can provide patients with real-time feedback and continuously assist them in filling their exercise prescription. It is worth noting that rehabilitation robots and assistive devices enable people with limited mobility to reacquire exercise capacity. In addition, an increasing number of exercise modalities have been reported to improve health. For example, some traditional Chinese exercise modalities, Qigong and Baduanjin, have been shown to combine physical practice with mental practice, and have multiple health benefits (Zou et al., 2018; Kuo et al., 2021; Yuen et al., 2021). These advances in technology and theory further facilitated the implementation of the “exercise is medicine” initiative.

Exercise is known as a form of medicine owing to its numerous health benefits. However, medicine inevitably has side effects, and exercise as a medicine is no exception. Sudden high-intensity exercise in the short term is associated with many risks. People, particularly the elderly, are susceptible to exercise-associated injuries and conditions without scientific guidance. In addition, there is a decline in immunity after a single bout of long-term and high-intensity exercise, rather than the general thought that exercise improves immunity (Simpson et al., 2020). This decline lasts for approximately 24 h, causing the immune system to respond poorly to foreign antigens and increasing the risk of acquiring an infection. Therefore, the rational use of exercise in medicine is very important. Avoiding exercise injuries and exercise-related diseases is the focus of exercise plan development.

There are three major principles for exercise, individualization, gradualness and persistence. Individualization means that people should choose the intensity and time of exercise according to their personal situation. Blindly following other people’s exercise experiences is not recommended. For gradualness, the intensity and time of exercise should be increased step by step. Sudden high intensity and prolonged exercise can pose harm to the body. The principle of persistence refers to that people should take exercise as a part of life and develop regular habits. The loss of adherence to exercise makes it difficult to achieve the expected results. Choosing the modality and the intensity of exercise is a complex task. However, it is worthy saying that regular exercise, regardless of type, duration, or intensity, is beneficial. Here we recommend running or brisk walking, which is the simplest form of exercise, 3–5 days a week, once a day, 45–60 min each time, in combination with appropriate resistance training. More importantly, the development of modules involved in the “exercise is medicine” initiative and the formation of normative medical work for exercise guidance need to be stimulated. Sustained efforts of establishing the “exercise is medicine” network are required to encourage more people to get involved in active health programs.

In conclusion, exercise is a form of medicine that deserves to be included in health plans. This is not only a concern in rehabilitation but has also become a universal health concern.

## Author contributions

JHL and HQ wrote the first draft. JNL designed and revised the manuscript. All authors approved the submitted version.
